# Dementia and Multimorbidity Trends in Al-Baha, Saudi Arabia: An Analytical Retrospective Study Using Records-Based Data

**DOI:** 10.7759/cureus.52507

**Published:** 2024-01-18

**Authors:** Saba Beigh, Remas Adnan, Al-Johrah Abdulaziz, Samia Abdullah, Nada Nasser, Rana Ghazzay, Ruba Abdulaziz, Ethar Mohammed, Rabab Morsy Ahmad, Mohammed Ali Alshehri

**Affiliations:** 1 Public Health, Albaha University, Al-Baha, SAU

**Keywords:** neurological disorders, parkinson’s disease, alzheimer’s disease, comorbidities, therapeutic regimen, dementia

## Abstract

Background: The prevalence of dementia is escalating significantly, posing a substantial societal burden. Currently, there exists a dearth of comprehensive health data about dementia patients in Saudi Arabia, particularly within Al-Baha City.

Methods: A retrospective case-series study was undertaken to ascertain the prevalence of dementia within the populace of the Al-Baha region, Kingdom of Saudi Arabia. This investigation utilized hospital-based records encompassing individuals exhibiting symptoms or diagnosed with dementia and its related forms across the Al-Baha region. Furthermore, the study aimed to evaluate the burden of comorbidities among dementia patients and document the pharmacological therapeutic interventions administered to manage dementia and its associated concurrent health conditions.

Results: Our investigation explored the prevalence rates of various forms of dementia and the accompanying comorbidities among affected individuals. The study spanned from August 2020 to August 2023. Our study encompassed 407 patients diagnosed with Alzheimer's disease (AD), Parkinson's disease, vascular dementia (VaD), or other forms of dementia who were either admitted to or attended tertiary hospitals in Al-Baha. Assessment of the comorbidity burden was conducted using the Charlson Comorbidity Index (CCI). Our findings revealed that among these patients, 13.3% presented with AD, 23.6% with VaD, 33.4% with Parkinson's disease, 15.75% with amnesia, and 14.0% with other types of dementia. The spectrum of comorbidities observed among dementia patients encompassed various conditions, with diabetes mellitus emerging as the predominant comorbidity (19.1%), followed by hypertension (16.4%). Additionally, manifestations of depression were noted in 14% of patients, while 9.82% suffered from paralysis. Chronic conditions such as cancer, chronic obstructive pulmonary disorder (COPD), and cervical spondylosis were also observed among individuals afflicted with dementia and its varied forms. Statistically significant correlations were established between gender, age, nationality, comorbidities, and the prevalence of dementia. Therapeutic interventions in the form of pharmacological treatments were prescribed for dementia patients with comorbidities. Commonly administered medications included Amlod (6.3%), Amlodipine (6.6%), Amlor (5.8%), Aspirin (10.5%), chemotherapeutic drugs (4.4%), Glipizide (8.5%), Lantus (11.3%), Levodopa (23.5%), Metformin (7.8%), acetylcholinesterase inhibitors (6.8%), and Pulmicort (7.86%). These medications aimed to alleviate symptoms associated with dementia and its accompanying comorbidities.

Conclusions: Our investigation underscores the substantial burden of comorbidities experienced by dementia patients. These findings offer crucial insights into the overall health status of individuals grappling with dementia, serving as a catalyst for increased awareness among clinicians and policymakers. Such awareness can drive improvements in medical care and support frameworks tailored to the specific needs of dementia patients.

## Introduction

As individuals advance in age, a growing sector of the elderly demographic grapples with dementia, recognized as the predominant neurodegenerative syndrome [1]. This condition precipitates a reduction in memory retention capabilities, coupled with a decline across multiple cognitive faculties [1]. Dementia inflicts significant harm upon patients, concurrently imposing a considerable financial strain on society at large [1].

Dementia, an increasingly prevalent neurodegenerative syndrome characterized by progressive memory loss and cognitive decline, impacts a growing elderly demographic. This condition imposes significant personal hardship on patients and exerts a substantial economic burden on society [2]. Typically marked by impaired planning abilities, memory loss, and difficulty executing daily tasks, dementia also adversely affects cognitive functions such as language comprehension, time perception, and spatial orientation [3]. Dementia encompasses a spectrum of over a hundred distinct types, each associated with specific patterns of brain cell damage tied to particular brain regions [4]. Elevated levels of certain proteins within and outside brain cells hinder cellular health and communication, contributing to conditions like Alzheimer's disease [5]. Initial damage often occurs in the hippocampus, which is crucial for memory and learning, leading to early symptoms of memory loss. Parkinson's disease, another neurodegenerative disorder, primarily affects dopamine-producing neurons in the substantia nigra, causing a gradual onset of symptoms over several years [6]. The manifestations of Parkinson's disease vary among individuals but commonly include tremors, limb rigidity, slow movement, and difficulties with gait and balance [6].

Alzheimer's disease remains the most prevalent form of dementia, often co-occurring with other types such as Lewy body dementia or vascular dementia [7]. However, there are limited clinical data on effectively treating patients dealing with multiple forms of dementia simultaneously. Moreover, individuals with Alzheimer's disease frequently exhibit a high prevalence of medical comorbidities like heart disease, diabetes, and various forms of cancer [8].

Slowing the progression of dementia involves mitigating risk factors and implementing cognitive training programs. In regions like the Middle East, where the prevalence of dementia is high but research is scarce, understanding its impact becomes essential. Limited data from specific studies in Saudi Arabia indicate varying prevalence rates for dementia, highlighting the need for more comprehensive research in this area [9]. Saudi Arabia's aging population is on the rise, with projections indicating a significant increase in individuals over 60 years old in the coming decades [10]. This demographic shift correlates with a higher likelihood of cognitive impairment as life expectancy increases. Globally, dementia prevalence is expected to triple in the next two decades, making research across societies crucial for understanding the disease's impact influenced by cultural nuances and technological advancements [11].

A significant number of elderly individuals admitted to hospitals for physical conditions also contend with dementia and related cognitive limitations [12]. However, accurately quantifying the exact number of patients affected by these health issues remains challenging at this time. Earlier research endeavors faced significant challenges in comparability due to widely varying methodologies and typically involved small, non-representative sample sizes. Consequently, these studies produced markedly divergent prevalence estimations, limiting their utility for effective healthcare planning concerning dementia [13]. Hospital stays often prove highly distressing for individuals grappling with both dementia and other health complications. Such circumstances can trigger rapid declines in cognitive and functional abilities [14]. Moreover, patients with comorbid dementia face approximately double the risk of institutionalization and mortality compared to those without cognitive impairments [15]. The dearth of comprehensive knowledge regarding the prevalence and distribution of cognitive disorders presents a barrier to delivering enhanced, tailored care that meets the specific needs of these patient cohorts [16].

The growing elderly population is poised to significantly influence healthcare and social service provisions. However, there exists limited information pertaining to dementia within the Saudi population. Comprehensive data regarding the demographics characterizing this group or the etiological diagnoses among affected individuals in Saudi Arabia remains scarce. Insufficient comprehensive data exists regarding dementia among the elderly in Saudi Arabia, notably within regions such as Al-Baha. This study seeks to investigate and assess the overall health status and comorbidity prevalence among individuals afflicted with diverse forms of dementia across various areas within the Baha Province of Saudi Arabia. Additionally, the research endeavors to examine the array of therapeutic medications administered to patients with various neurological disorders, aiming to bridge the existing knowledge gap in this domain.

## Materials and methods

Participants

To investigate concurrent medical conditions among individuals with dementia, a comprehensive retrospective survey was conducted across five regions in Baha Province: Bhaljureshi, Al-Mandaq, Al-Baha, Banisar, and Al-Hajrah. The primary objective of this investigation was to compile exhaustive case histories of dementia patients. This retrospective case-series study gathered secondary data from medical institutions in the Al-Baha region, which included King Fahad Hospital, Eradah Complex for Mental Health (Psychiatric Hospital Bhaljureshi), and Prince Mishari Hospital. Demographic characteristics, pharmaceutical utilization, and comorbid conditions were systematically documented through a scrupulous examination of medical records retrieved from these tertiary hospitals. The study cohort encompassed individuals aged 40 years and above diagnosed with various forms of dementia, including Alzheimer's disease, vascular dementia, Parkinson's disorder, or other categorically unspecified types. The investigation focused on individuals admitted to hospitals between August 2020 and August 2023, all diagnosed with diverse dementia subtypes. Diagnosis of dementia subtypes was meticulously performed by seasoned neurologists, employing comprehensive medical examinations and neuropsychological evaluations in strict adherence to globally recognized diagnostic criteria, as exemplified by the National Institute of Neurological and Communicative Disorders and Stroke-Alzheimer's Disease and Related Disorders Association guidelines. The study strategically prioritized the extraction of secondary data from patient databases, with a specific focus on those diagnosed with neurological disorders. Eligible participants were required to possess a confirmed clinical diagnosis of dementia, delineated by specified subtypes and severity levels. Additional inclusion criteria mandated participants to fall within a designated age range, exhibit proficient language skills, maintain stable medication regimens, and possess the capacity to provide informed consent or have a legally authorized representative. Diversity considerations and, when applicable, inclusion of caregivers or family members were duly addressed. Certain exclusions were instituted, precluding patients admitted to the emergency department, intensive care unit, and coronary care unit from participation.

**Figure 1:**
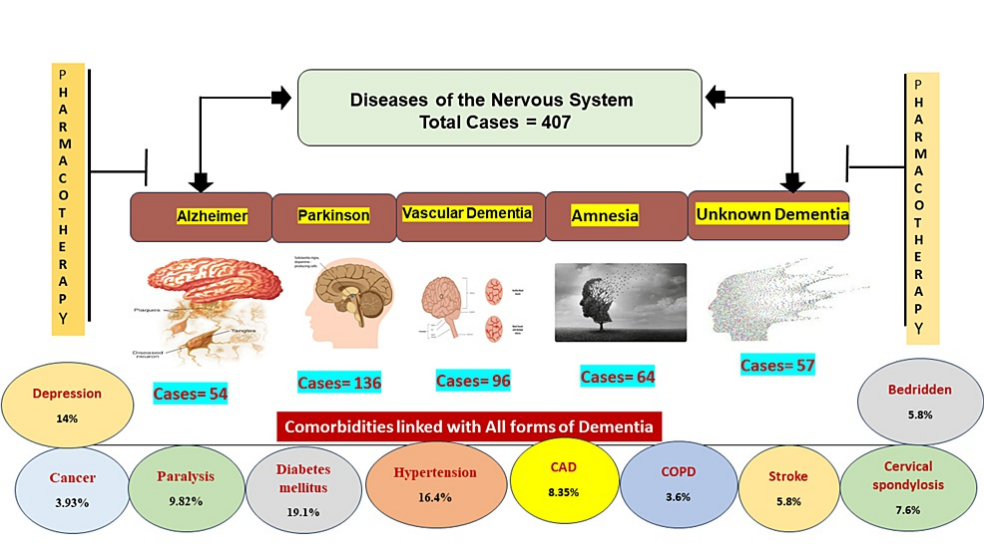
A graphical depiction illustrating dementia, its classifications, and the study design examining its coexisting health conditions.

Moreover, individuals with psychotic and mood disorders admitted to the psychiatric ward were systematically omitted from the study. Stringent eligibility criteria further ensured that participants had not undergone surgery within the preceding 8 hours, did not meet delirium criteria outlined in the Diagnostic and Statistical Manual of Mental Disorders, Fifth Edition (DSM-5), and did not manifest altered levels of consciousness or acute neurological or metabolic conditions. Ethical clearance for the study was diligently obtained from the Institutional Review Boards of Al-Baha University, affirming the adherence to rigorous ethical standards in the conduct of the research. The visual illustration depicted in Figure 1 exemplifies the graphical portrayal derived from our retrospective study's findings.

Data retrieval

All demographic and clinical information, encompassing gender, age, nationality, geographic region, treatment protocols, and comorbidities-such as hypertension, dementia, depression, diabetes mellitus, cancer, and other conditions accounted for within the Charlson Comorbidity Index (CCI)- was gathered. In this investigation, a cohort comprising 407 individuals was identified with distinct neurological disorders, some of whom necessitated hospitalization due to severe concurrent ailments coinciding with their neurological conditions. Diagnoses covered a spectrum, including Parkinson's and Alzheimer's diseases, short-term memory impairment, and unspecified forms of dementia. All relevant demographic and clinical information, such as name, gender, age, marital status, nationality, location, disease initiation year, neurological conditions, and health issues like hypertension, dementia, depression, diabetes, and cancer, computed in the CCI were extracted from hospital databases. Table 1 shows the CCI index of comorbidities based on severity.

**Table 1:** CCI scores of various comorbidities CAD: Coronary artery disease; COPD: Chronic obstructive pulmonary disease; CCI: Charlson Comorbidity Index

Comorbidity	Score
Bedridden	1
CAD	1
Cancer	2
Cervical spondylosis	1
COPD	1
Depression	1
Diabetes mellitus	1
Hearing loss	1
Hypertension	1
Paralysis	1
Stroke	2

The assessment of chronic comorbidity severity was determined through the Charlson Co-Morbidity scale, a metric comprising 19 diseases weighted according to their correlation with mortality rates. Meanwhile, the Acute Physiology and Chronic Health Evaluation (APACHE II) system gauges the severity of acute illness by considering 12 standard physiological parameters, such as core temperature, respiratory rate, mean arterial pressure, Glasgow Coma Scale, and laboratory metrics like serum sodium, potassium, creatinine, and hematocrit levels. Subsequently, the acquired data were transferred into Excel and SPSS software for analysis.

Ethical approval and informed consent

The study was conducted according to the guidelines of the Declaration of Health Affairs and approved by the Institutional Review Board of Al-Baha University (IRB number: KFH/IRB23052023/9)

Patient and Public Participation 

There was no involvement of patients or the general public.

Data analysis

Statistical analysis was executed utilizing the SPSS/PC statistical software, wherein chi-square tests were employed to evaluate variances in demographic characteristics, comorbidities, and types of dementia, adhering to a significance level established at p < 0.05.

## Results

Characteristics of participants

Within the entirety of 407 patients under observation, the gender distribution revealed 224 individuals identifying as female, constituting 55% of the total cohort, while the male population accounted for 183 patients, representing 45%. Across various age cohorts, a spectrum of neurological disorders was observed. Notably, the range of affliction spanned from the youngest patient at 41 years to the oldest at 114 years, as delineated in Table 1. Among the 407 patients surveyed, 12 fell within the 40-60 age bracket, constituting 2.9% of the total. Within the 61-80 age bracket, 126 patients, accounting for 31% of the cohort, were found to suffer from neurological disorders.

Furthermore, there were 245 patients aged 81-100 and 24 patients aged 101-120, comprising 5.9% of the total patients in the latter group. The average age within the specified age group was determined to be 74.7 years, with a standard deviation of 12.5 years, denoting the variability of ages around this mean value (74.7 ± 12.5). In our investigation, we observed that among the cohort of patients examined, there were 268 individuals classified as married, representing 65.8% of the total sample. Among this group, a significant prevalence of various neurological disorders was identified. Additionally, the cohort included 130 divorced individuals, constituting 31.9% of the sample, showcasing a noteworthy incidence of neurological disorders. Notably, individuals categorized as being in a single marital status comprised the smallest group, numbering at a mere 2.2% in contribution to the occurrence of neurological disorders within this study. Among the populations examined, the Saudi demographic displayed the highest incidence of various neurological disorders, totaling 371 cases, which represented 91.2% of the studied cohort.

Conversely, the non-Saudi population exhibited notably fewer instances, with only 36 cases constituting a mere 8.8% of the observed occurrences of neurological disorders. In our study, encompassing patients from five regions within the Baha province, the highest number of patients, totaling 290 individuals, accounting for 71.3% of the total, originated from Bhaljureshi. Subsequently, the region of Al-Mandaq contributed 56 patients, representing 13.8% of the cohort.

Additionally, 53 patients, comprising 13% of the sample, hailed from the Al-Baha region. In contrast, a smaller number of patients, specifically 6 individuals (1.4%), were identified from Banisar, while the fewest patients, totaling 2 individuals (0.5%), were associated with Al-Hajrah within the Baha province. Table 2 illustrates the distribution of various severe neurological disorders among dementia patients, indicating that 33.4% of these individuals experienced concomitant Parkinson's disorder. Vascular dementia was observed in 23.6% of the cases, while amnesia was in 15.7% of the dementia patients. Furthermore, Alzheimer's disease was identified in 13.3% of the cases, with the remaining 14% attributed to other forms of dementia. Dementia patients exhibited a spectrum of concurrent medical conditions, with prevalent comorbidities including diabetes, accounting for 19.1% of cases, followed by hypertension at 16.4%, and depression at 14%. Paralysis was identified as a comorbidity in 9.82% of cases, succeeded by coronary artery disease affecting 8.35% of individuals. Additionally, cervical spondylosis was discernible in 7.6% of dementia cases. Table 2 illustrates further comorbidities present among dementia patients.

**Table 2:** Basic attributes of individuals in the study impacted by dementia N = number of individuals in the study %= percentage of individuals in the study; SD = standard deviation; CAD: Coronary artery disease

Variable categories	Total Cases = 407	
Age	N	%
40-60Y	12	2.9
61-80Y	126	31.0
81-100Y	245	60.2
101-120Y	24	5.9
Mean ± SD 74.7 ± 12.5		
Gender	N	%
Female	224	55.0
Male	183	45.0
Marital status	N	%
Divorced	130	31.9
Married	268	65.8
Single	9	2.2
Nationality	N	%
Saudi	371	91.2
Non-Saudi	36	8.8
Region	N	%
Bhaljureshi	290	71.3
Al-Mandaq	56	13.8
Al-Baha	53	13.0
Banisar	6	1.4
Al-Hajrah	2	0.5
Diagnosis	N	%
Alzheimer's disease	54	13.3
Vascular Dementia	96	23.6
Parkinson's disease	136	33.4
Amnesia	64	15.7
Other Dementia	57	14.0
Year of Disease onset	N	%
2020	41	10.1
2021	156	38.3
2022	119	29.2
2023	91	22.4
Other Comorbidities	N	%
Cancer	16	3.93
Paralysis	40	9.82
Cervical spondylosis	31	7.6
Hearing loss	21	5.1
Hypertension	67	16.4
Depression	57	14
Diabetes mellitus	78	19.1
CAD	34	8.35
Bedridden	24	5.8
COPD	15	3.6
Stroke	24	5.8
Treatment Regimen	N	%
Levodopa	96	23.5
Amlod	26	6.3
Glizide	35	8.5
Metformin	32	7.8
Amlodipine	27	6.6
Acetylcholinesterase inhibitors	28	6.8
Amlor	24	5.8
Lantus	46	11.3
Pulmicort	32	7.86
Aspirin	43	10.5
Chemotherapeutic drugs	18	4.4

Further we conducted an analysis of mixed comorbidities in both male and female patients. Table 3 provides a comprehensive overview of these mixed comorbidities. A total of 160 patients were identified who suffered from Hypertension, Diabetes Mellitus, Paralysis, and dementia simultaneously. Among these, 44.8% were males, and 34.8% were females. Additionally, 69 patients with dementia experienced mixed comorbidities, including diabetes mellitus, paralysis, coronary artery disease (CAD), and stroke. In this group, 18.5% were females, and 15.6% were males. Furthermore, 66 patients exhibited mixed comorbidities involving hypertension, diabetes mellitus, paralysis, cancer, and depression. Among them, 18.3% were females, and 13.6% were males. Moreover, we identified 56 patients (15.6% females and 11.47% males) with mixed comorbidities comprising Hypertension, Diabetes Mellitus, Paralysis, and Chronic Obstructive Pulmonary Disease (COPD). Lastly, 56 patients (15.6% females and 11.47% males) were diagnosed with mixed comorbidities involving cervical spondylosis, hearing loss, depression, and bedridden status.

**Table 3:** Displays the occurrence and proportion of mixed comorbidities in both male and female individuals diagnosed with dementia Illustrates the count (N) and percentage (%) of male and female patients, with "DM" denoting Diabetes Mellitus, "COPD" representing Chronic Obstructive Pulmonary Disorders, and "CAD" standing for Coronary artery disease

Mixed Comorbidities	Male- N (%)	Female - N (%)	Total
DM + Paralysis + CAD + stroke	34 (15.6%)	35 (18.5%)	69
Hypertension + DM + Paralysis	82 (44.8%)	78 (34.8%)	160
Hypertension + DM + Paralysis + COPD	21 (11.47%)	35 (15.6%)	56
Hypertension + DM + Paralysis + Cancer + Depression	25 (13.66%)	41 (18.3%)	66
Cervical spondylosis + Hearing loss + Depression + Bedridden	21 (11.47%)	35 (15.6%)	56

The association of neurological disorders with gender, age, and comorbid conditions

Our inquiry delved into disparities in the incidence rates of diverse dementia types among patients, considering gender, age, and comorbidity factors, as shown in Table 4. Specifically, our findings indicated a higher prevalence of Alzheimer's disease among females in contrast to males, while amnesia/short-term memory loss exhibited a predominant occurrence among males as opposed to females. Parkinson's disorder manifested prominently in both genders, with 74 instances among females and 62 among males. Vascular disorder, conversely, was solely identified in females, with 96 cases of vascular dementia observed in this gender group. Additionally, our study identified 52 instances of other types of dementia exclusively in males. Statistical analysis revealed a statistically significant difference of 0.001 among various neurological disorders with gender with χ2 =270.675. The neurological disorders were also examined in relation to various age brackets within the patient population (Table 4). Our study unveiled 42 instances of Alzheimer's disease among individuals aged 61-80 years and 12 cases within the 40-60 years age bracket.

Interestingly, no instances of Alzheimer's were observed in the age groups of 81-100 years or 101-120 years. Amnesia/short-term memory loss was solely detected within the age group of 81-100 years. The highest incidence of Parkinson's disease was identified among individuals aged 81-100 years. Vascular dementia cases numbered 84 among those aged 61-80 years and 12 cases among the 81-100 years age group. Additionally, our study uncovered 33 instances of unidentified dementia within the 81-100 years age range. Statistical analysis revealed a statistically significant difference of 0.001 among various neurological disorders with age. Our investigation explored the correlation between various forms of dementia and the presence of diverse comorbidities among patients. Within the spectrum of dementia, we observed specific associations as Sixteen individuals diagnosed with Alzheimer's disease concurrently experienced cancer, while 38 exhibited paralysis as comorbidity. Among patients with amnesia or short-term memory loss, six were bedridden, thirty-four had coronary artery disease, and twenty-four were diagnosed with diabetes mellitus. Within the Parkinson's disease cohort, fifty-seven patients presented with depression, fifty had diabetes mellitus, and twenty-five suffered from hypertension. Among those diagnosed with Vascular dementia (VaD), twenty-one had hearing impairment, forty-two had hypertension, and two exhibited paralysis. Patients with other forms of dementia showcased additional comorbidities: eighteen were bedridden, fifteen had chronic obstructive pulmonary disorder (COPD), and twenty-four experienced strokes. A statistically significant difference of 0.001 and a chi-square value of χ2 = 1276.147 was identified among the various types of dementia patients concerning the comorbidities they were experiencing (Table 4). Further, Table 5 illustrates the matrix displaying correlations among study variables, accompanied by significance tests corrected for multiple comparisons using Holm's Method.

**Table 4:** Various Neurological Disorders Vs Gender, Age, Comorbidities χ2= chi-square analysis. Significance difference is indicated by “S” and non-significance by “NS”. CAD: Coronary artery disease; COPD: Chronic obstructive pulmonary disease

Neurological Disorders and Gender						
Disorders / Gender	Alzheimer Disorder	Amnesia	Other Disorder	Parkinson disorders	Vascular disorder	χ^2^ P value
Male	0	64	57	62	0	270.675 P < 0.001^s^
Female	54	0	0	74	96	
Neurological Disorders with Age						
Disorders / Age	Alzheimer Disorder	Amnesia	Other Disorder	Parkinson disorders	Vascular disorder	χ^2^ P value
40-60Y	12	0	0	0	0	564.223 P < 0.001^s^
61-80Y	42	0	0	0	84	
81-100Y	0	64	33	136	12	
101-120Y	0	0	24	0	0	
Neurological Disorders with Comorbidities						
Disorders / Comorbidities	Alzheimer Disorder	Amnesia	Other Disorder	Parkinson disorders	Vascular disorder	χ^2^ P value
Bedridden	0	6	18	0	0	1276.147 P < 0.001^s^
CAD	0	34	0	0	0	
Cancer	16	0	0	0	0	
Cervical spondylosis	0	0	0	0	31	
COPD	0	0	15	0	0	
Depression	0	0	0	57	0	
Diabetes mellitus	0	24	0	54	0	
Hearing loss	0	0	0	0	21	
Hypertension	0	0	0	25	42	
Paralysis	38	0	0	0	2	
Stroke	0	0	24	0	0	

**Table 5:** Matrix Displaying Correlations Among Study Variables with Significance Tests Corrected for Multiple Comparisons using Holm's Method

Pearson Correlation between variables	Age	Year of disease onset	Gender	Marital	Nationality	Region	Diagnosis	Comorbidities	Treatment
Age	1								
Year of disease onset	-0.061	1							
Gender	0.058	-0.148^**^	1						
Marital	-0.054	0.037	0.228^**^	1					
Nationality	0.016	0.120^*^	0.003	0.114^*^	1				
Region	-0.159^**^	0.139^**^	0.041	0.390^**^	0.110^*^	1			
Diagnosis	-0.040	-0.136^**^	0.029	0.104^*^	-0.031	-0.021	1		
Comorbidities	-0.116^*^	0.019	0.046	0.160^**^	-0.033	0.124^*^	0.082	1	
Treatment	-0.044	0.027	0.095	-0.038	0.021	-0.077	0.088	-0.033	1
**. Correlation is significant at the 0.01 level (2-tailed).									
*. Correlation is significant at the 0.05 level (2-tailed).									

Correlation between comorbidities and various demographic variables on the prevalence of a disease

Table 6 displays the comparison of various comorbidities across gender, nationality, year of disease onset, and region. A significant distinction (χ2=379.209 and p<0.001) was noted concerning the gender distribution among patients." Among dementia patients, diverse comorbidities were observed across genders. The most prevalent comorbidity in male dementia cases was diabetes mellitus, noted in 78 individuals. Conversely, hypertension was predominant among female dementia patients, affecting 67 individuals while being absent in males. Paralysis was exclusively seen in 40 female dementia patients, with no recorded cases in males.

**Table 6:** Various comorbidities vs. gender, nationality, year of disease onset, age and region χ2= chi-square analysis. Significance difference is indicated by “S” and non-significance by “NS”

Various Comorbidities vs. Variables												
Comorbidities	Bedridden	CAD	Cancer	Cervical Spondylosis	COPD	Depression	Diabetes Mellitus	Hearing Loss	Hypertension	Paralysis	Stroke	χ^2^ P value
Comorbidities and Gender												
Male	24	34	0	0	15	8	78	0	0	0	24	379.209
Female	0	0	16	31	0	49	0	21	67	40	0	P < 0.001^s^
Comorbidities and Nationality												
Saudi	20	30	16	31	11	47	78	21	59	40	18	40.051 P < 0.001^s^
Non-Saudi	4	4	0	0	4	10	0	0	8	0	6	
Comorbidities and Year of onset												
2020	0	5	1	4	0	5	10	1	8	6	1	30.657 P > 0.432 ^ns^
2021	9	10	2	14	4	20	36	12	22	18	9	
2022	9	12	8	7	8	18	16	3	22	9	7	
2023	6	7	5	6	3	14	16	5	15	7	7	
Comorbidities with Age												
40-60Y	0	0	12	0	0	0	0	0	0	0	0	1028.523 P < 0.000^s^
61-80Y	0	0	4	31	0	0	0	21	30	40	0	
81-100Y	24	34	0	0	15	57	78	0	37	0	0	
101-120Y	0	0	0	0	0	0	0	0	0	0	24	
Comorbidities with Region												
Banisar	0	0	0	1	0	0	3	1	0	1	0	167.463 P < 0.001^s^
Al-Baha	0	16	0	1	9	1	13	1	1	3	8	
Al-Hajrah	0	0	0	0	0	0	0	1	0	1	0	
Al-Mandaq	11	4	0	0	4	8	5	1	16	2	5	
Bhaljureshi	13	14	16	29	2	48	57	17	50	33	11	

Additionally, coronary artery disease affected 34 male dementia patients more so than females. Cervical spondylosis was evident in 31 female dementia patients, while depression was observed in 49 females and 8 males. Stroke exhibited the highest incidence among male dementia patients, with 24 cases, surpassing female occurrences. Detailed gender-specific comorbidity data is provided in Table 6." A notable differentiation (χ2=379.209 and P<0.001) was identified in gender-specific comorbidities, signifying statistical significance. Our ongoing investigation noted a fluctuation in the comorbidity load throughout the study duration. Specifically, during the 2021 period, the maximum number of dementia patients-totaling 156 cases were admitted, showcasing a higher burden of various comorbidities. Subsequently, in 2022, 119 dementia patients exhibited comorbidities, followed by a reduced count of 91 cases in 2023. Notably, the least comorbidity burden was observed in 2020, with only 41 dementia patients displaying additional health conditions.

Interestingly, upon statistical analysis using the chi-square test, we did not find any significant differences between the comorbidity burden and the respective years, although the chi-square value was not reported ((χ2=30.657 and P<0.432ns). The current investigation encompassed dementia patients hailing from various areas within the Baha province situated in Saudi Arabia. Predominantly noted among these patients were comorbidities linked to diabetes mellitus, notably prevalent in the Bhaljureshi region. This prominence aligns with our findings, indicating the highest frequency of comorbidities among dementia patients residing in the Bhaljureshi area, potentially attributed to the elevated number of reported dementia cases within this region. In contrast, the Al-Manqad and Al-Baha regions exhibited a moderate incidence of dementia cases.

Conversely, the regions of Banisar and Al-Hajra displayed the lowest occurrences of comorbidities among the studied dementia patients. A statistically significant difference of 0.001 was identified among the diverse types of comorbidities observed across the various regions studied within Baha. Although the specific chi-square value was 167.463, this finding suggests a notable association between different comorbidities and the distinct geographic regions within the Baha province. As highlighted by our study findings, the prevalence of comorbidities notably escalates with advancing age. Within the age bracket of 81-100 years, a heightened comorbidity burden was observed, particularly in cases of diabetes mellitus and depression. In the 61-80 years age group, the prevalence of cervical spondylosis, paralysis, and hypertension was notably prominent.

Interestingly, among individuals aged 40-60 years, severe comorbidity was predominantly associated with cancer, with minimal occurrences of other comorbidities. For those aged 101-120 years, a notable prominence of stroke cases was observed. Significantly, a p-value of 0.001 indicated a substantial difference between the various types of comorbidities and distinct age groups with a chi-square of 1028.523.

"Tailored Therapeutic Approaches for Diverse Dementia Subtypes and Comorbidities Across Diverse Demographics and Geographies

Our analysis observed a statistically significant variance with a P-value of 0.001 and a chi-square value of 2.34 when examining the diverse therapeutic medications administered to patients affected by different forms of dementia and concurrent comorbidities. The research findings revealed specific medication patterns: Levodopa was notably prescribed to patients diagnosed with Alzheimer's subtype dementia. For individuals experiencing amnesia in conjunction with other comorbidities, a combined therapeutic approach involving Aspirin, Lantus, and Pulmicort was recommended. Likewise, patients with unspecified dementia types were treated with a regimen of Aspirin, Lantus, and Pulmicort due to their concurrent comorbidities, addressing both dementia and their other health conditions. Those diagnosed with Parkinson's disease (PD) subtype dementia were provided with a combination regimen encompassing Amlodipine, Amlor, Glipizide, Metformin, Lantus, and Paracetamol to manage both dementia symptoms and additional comorbidities.

Meanwhile, patients with vascular dementia (VaD) subtype were prescribed a combination regimen involving Amlod, Glipizide, and Levodopa to address VaD alongside their concurrent comorbidities. The therapeutic regimens prescribed for different subtypes of dementia, in conjunction with the accompanying comorbidities, were juxtaposed with various geographical and demographic variables such as region, nationality, age, and gender. These comparisons have been detailed in Table 7.

**Table 7:** The variance in dementia subtypes, their associated comorbidities, and their distribution across diverse demographic and geographic profiles was explored and analyzed. AD: Alzheimer’s disease, AM: Amnesia/short term memory loss, OD: Other dementia, PD: Parkinson’s disease, VaD: Vascular dementia, F: Female, M: Male. χ2= chi-square analysis, s= significance and ns represent non-significance.

The correlation between the treatment regimen administered and the quantity of accompanying disorders												
Disorders	Amlod	Amlodipine	Amlor	Aspirin	Anticancer drugs	Glipizide	Lantus	Levodopa	Metformin	Acetylcholinesterase inhibitors	Pulmicort	χ^2^ P value
AD	0	0	0	0	0	0	0	54	0	0	0	1157.835 P< 0.001^s^
AM	0	0	0	4	0	0	28	0	0	0	32	
OD	0	0	0	39	18	0	0	0	0	0	0	
PD	0	27	24	0	0	7	18	0	32	28	0	
VaD	26	0	0	0	0	28	0	42	0	0	0	
The relationship between the treatment protocols and specific geographic regions												
Region	Amlod	Amlodipine	Amlor	Aspirin	Anticancer drugs	Glipizide	Lantus	Levodopa	Metformin	Acetylcholinesterase inhibitors	Pulmicort	χ^2^ P value
Banisar	1	0	0	0	0	0	2	2	0	0	0	170.375 P< 0.001
Al-Baha	1	0	0	9	8	0	14	5	1	3	12	
Al-Hajrah	1	0	0	0	0	0	0	1	0	0	0	
Al-Mandaq	0	5	0	16	4	5	2	3	14	3	4	
Bhaljureshi	23	22	24	18	6	30	28	85	17	21	16	
The association between nationality and the treatment administered												
Nationality	Amlod	Amlodipine	Amlor	Aspirin	Anticancer drugs	Glipizide	Lantus	Levodopa	Metformin	Acetylcholinesterase inhibitors	Pulmicort	χ^2^ P value
Non-Saudi	0	6	0	8	6	5	0	0	7	0	0	54.360 P< 0.001^s^
Saudi	26	21	24	35	12	30	46	96	25	28	26	
The correlation between age demographics and the treatment provided												
Age	Amlod	Amlodipine	Amlor	Aspirin	Anticancer drugs	Glipizide	Lantus	Levodopa	Metformin	Acetylcholinesterase inhibitors	Pulmicort	χ^2^ P value
40-60Y	0	0	0	0	0	0	0	12	0	0	0	692.344 P< 0.001^s^
61-80Y	26	0	0	0	0	16	0	84	0	0	0	
81-100Y	0	27	24	37	0	19	46	0	32	28	32	
101-120Y	0	0	0	6	18	0	0	0	0	0	0	
The correlation between Gender demographics and the treatment provided												
Gender	Amlod	Amlodipine	Amlor	Aspirin	Anticancer drugs	Glipizide	Lantus	Levodopa	Metformin	Acetylcholinesterase inhibitors	Pulmicort	χ^2^ P value
F	26	27	0	0	0	35	0	96	32	8	0	383.909 P< 0.001^s^
M	0	0	24	43	18	0	46	0	0	20	32	

## Discussion

In an extensive study conducted from August 2020 to August 2023, encompassing a cohort of 407 dementia patients admitted to two major hospitals in distinct regions of Baha province in Saudi Arabia, and our investigation produced significant findings. Our study revealed notable positive associations between the Charlson Comorbidity Index (CCI) and age across all dementia types. Particularly, Parkinson's disease (PD) patients exhibited higher CCI scores compared to patients with Alzheimer's disease (AD) or vascular dementia (VaD). Moreover, we observed a higher CCI trend in females than males.

The escalation in dementia inpatient numbers over time indicates improved access to appropriate treatment. This rise may stem from enhanced quality of life, increased medical awareness, or a demographic shift towards an aging Saudi Arabian population, where dementia prevalence is higher. Aligning with previous research, the mean age of PD patients surpassed that of other dementia types, underscoring age as a significant PD risk factor [17]. According to earlier research, Parkinson's disease stands as the most prevalent movement disorder in Saudi Arabia [18]. Anxiety and depression rank among their frequent non-motor symptoms. Several factors may contribute to this observed trend. In Saudi Arabia, individuals with mental health conditions often encounter insufficient care, potentially due to the stigma surrounding such disorders [19].

Interestingly, after adjusting for age and gender, PD patients displayed a higher CCI, suggesting a greater burden of severe comorbidities compared to other dementia subtypes. In Parkinson's disease, various additional health conditions play a role in hospitalizations, notably for pneumonia, trauma, infections, and psychiatric reasons. Individuals with chronic obstructive pulmonary disease (COPD), a history of stroke, peripheral vascular issues, or peptic ulcer disease face an elevated risk of hospitalization in connection to their Parkinson's condition [20]. Our study highlighted that a significant number of patients diagnosed with Parkinson's disease also presented depression as a major comorbidity alongside their primary condition.

Further earlier research findings unveiled that Parkinson's disease commonly affects older individuals [21], and consequently, age-related additional health conditions are often concurrent [21]. Among individuals with PD, the cumulative 5-year occurrence rates were 77% for osteoarthritis, 50% for ischemic heart disease, 33% for cancer, and 30% for diabetes mellitus [22]. As expected, our cohort exhibited an increase in CCI with age. Aging in individuals, particularly the elderly, is associated with gradual organ decline and continued physiological deterioration, leading to an augmented comorbidity burden among dementia patients.

Clinical and molecular investigations provide compelling evidence supporting the potential linkage between chronic ailments, such as diabetes, cardiovascular disease, depression, and inflammatory bowel disease, and elevated susceptibility to dementia and its various subtypes across diverse populations [23]. The conjecture posits that disruptions within several interrelated biological pathways may underlie the association between Alzheimer's disease (AD) and these concurrent health conditions. Notably, inflammation emerges as a shared dysregulated pathway, unifying a majority of the comorbidities affiliated with neurological disorders, including Alzheimer's disorder [24].

Consistent with prior studies, dementia patients were significantly older than individuals with other neurological disorders, emphasizing age as a primary risk factor. Females tend to receive PD diagnoses more frequently [25], consistent with our findings of a higher prevalence of PD and VaD among females in Saudi Arabia. Two gender-related trends were noted: firstly, higher hospitalization rates among females in Saudi Arabia, likely attributed to traditional gender roles and physiological changes post-childbirth; secondly, higher CCI among females, possibly due to increased prevalence of common diseases. Our study revealed differing comorbidity profiles between male and female dementia patients, with higher rates of various diseases in both genders, especially female patients.

Recent research in Saudi Arabia has highlighted the rising prevalence of diabetes among the elderly, a known risk factor for dementia, especially vascular dementia [26]. Research indicates that type 2 diabetes can heighten the susceptibility to conditions like Alzheimer's disease, vascular dementia, and other forms of cognitive decline. The shared cardiovascular issues that elevate the risk of type 2 diabetes also contribute to an increased vulnerability to dementia [27]. In alignment with our study, a substantial proportion of dementia patients were identified as having diabetes, establishing it as a significant comorbidity within the elderly population. There's substantial evidence indicating that elevated blood pressure heightens the risk of dementia [28]. The World Health Organization has recently classified hypertension and type 2 diabetes (T2D) as modifiable comorbidities that contribute to the development of dementia and Alzheimer's disease [29]. Long-term research studies have consistently shown that individuals with high blood pressure between the ages of 40 and 64 have a higher likelihood of developing dementia in their later years, especially vascular dementia [30]. Our study yielded similar outcomes. We observed a notable increase in hypertension among patients with vascular dementia, followed closely by those diagnosed with Parkinson's disease. Our study revealed a significant rise in hypertension among patients with dementia, emerging as a prominent comorbidity. Additionally, our study unveiled that among other types of unidentified dementia, a significant number of patients also presented with stroke as a noteworthy comorbidity.

Indeed, various studies have established a bidirectional relationship between stroke and dementia, where each serves as a risk factor for the other [31]. Data from some studies indicate that approximately 10 percent of individuals who have experienced a stroke will develop dementia within the initial year following the stroke [32]. Chronic obstructive pulmonary disease (COPD) was also observed to increase dementia risk, possibly due to shared mechanisms of neuronal damage with Alzheimer's disease. Moreover, elevated COPD levels among dementia patients may exacerbate the risk of other neurological disorders [33]. Notably, around 10% of dementia patients in our study suffered from cancer, higher than in the broader East Asian population, necessitating further investigation into the etiology of cancer development in neurological disorder patients [33]. In our study, we observed instances where Alzheimer's patients also presented with occurrences of cancer as a comorbid condition. Our findings reinforce this idea as the high-risk group we identified showed a heightened incidence of various cancers, known to escalate in risk with age. This suggests a potential for a more significant or possibly even an etiological relationship among these diverse comorbidities. Reports indicate that a majority of dementia patients experience other comorbidities, including paralysis, spondylitis, and being bedridden [34]. Our research findings aligned with this observation, revealing that dementia patients often had cervical spondylitis and paralysis as accompanying comorbidities.

Reports indicated the utilization of pharmacotherapies aimed at addressing diverse forms of dementia as well as concurrent health conditions [35, 36]. In another pivotal study conducted by Brown et al. in 2017, noteworthy correlations were highlighted between the utilization of primary care services and the volume of prescriptions [37]. Promoting widespread awareness about dementia is crucial to fostering understanding, empathy, and support for individuals affected by this condition. In a compelling study conducted by Aldharman et al in 2023, a noteworthy aspect is highlighted, asserting that 'An Assessment of Dementia Knowledge' significantly contributes to the understanding of cognitive health. This study underscores the critical importance of promoting widespread awareness about dementia [38]. Therefore, this finding adds valuable complexity to the ongoing conversation. This study illuminated substantial disparities in the utilization of medications for concurrent health conditions among individuals afflicted with different types of dementia. Our discovery revealed that individuals diagnosed with Alzheimer's disease commonly received acetylcholinesterase inhibitors, while those experiencing amnesia were often prescribed antidiabetic medication alongside corticosteroids in addition to their regular treatment regimen. Additional medications targeting various comorbidities were also prescribed, aiming to provide relief and address concurrent health issues experienced by the patients. In some studies, various pharmaceutical agents commonly prescribed for individuals grappling with diabetes and cardiovascular disease exhibit promising outcomes in patients affected by dementia [39]. Studies rooted in systems-based biology have identified common genetic factors and perturbed pathways, offering insights into the potential mechanistic foundations for the observed relationships among comorbid disorders in dementia [40].

Nevertheless, a comprehensive understanding of the precise mechanisms governing the occurrence of disease comorbidities in dementia, including its various subtypes, remains elusive. Our study is specifically concentrated on delineating the types of dementia afflicting patients and elucidating the emergence of comorbidities during the course of dementia. Our emphasis leans toward pharmacotherapy, analyzing and detailing the medication treatments involved in managing these conditions. Overall, our study offers valuable insights into the disease burden of dementia patients in Al-Baha, serving as a foundation for future comparative research and informing healthcare policies to improve patient care and overall quality of life for those with neurological disorders. These findings underscore the importance of prioritizing dementia care in acute hospitals, particularly cognitive screening for older individuals upon hospital presentation.

While our extensive study encompassed the demographic of dementia patients across diverse Al-Baha regions for three years, its retrospective design bears intrinsic limitations that could introduce selection bias. One major constraint is rooted in the study's retrospective nature, where methodologies such as cluster random selection for hospital recruitment were not utilized. The recruitment process solely relied on hospitalized patients, potentially skewing the sample towards individuals with advanced dementia or underlying health conditions. Moreover, due to the retrospective nature, evaluating inter-rater agreement was unfeasible within the confines of this investigation. As a result, the findings should be interpreted as indicative of prevalent comorbidities and hospital burdens among hospitalized dementia patients rather than precise incidence or prevalence rates representing the larger dementia populace.

Additionally, the absence of a control group matched for sex and age from the general population poses limitations in presenting an unbiased overview of comorbidity burdens among typical dementia patients. Another substantial constraint lies in accurately diagnosing specific dementia subtypes without access to biomarkers at the time of the study. Despite selecting tertiary hospitals renowned for accurate disease diagnosis, technological limitations persisted in subtype identification.

Recommendations

Some general recommendations that may help in caring for such patients: Develop individualized care plans for each patient, considering their specific type of dementia, comorbidities, and personal preferences. Establish a multidisciplinary healthcare team involving neurologists, geriatricians, nurses, and other specialists to provide comprehensive care. Schedule regular medical assessments to monitor both dementia progression and comorbidities. Adjust treatment plans as needed. Carefully manage medications, considering potential interactions and side effects. Regularly review the medication regimen to ensure appropriateness. Provide activities that stimulate cognitive function, such as puzzles, games, and reminiscence therapy, tailored to the individual's cognitive abilities. Establish a consistent daily routine, as familiarity and routine can help dementia patients feel more secure and reduce anxiety. Adapt the living environment to enhance safety and reduce confusion. Remove potential hazards, use clear signage, and maintain a calm and quiet atmosphere. Ensure proper nutrition by offering easy-to-eat, nutritious meals. Address any specific dietary needs related to comorbidities. Monitor and encourage proper hydration, especially if the patient has comorbid conditions that may impact fluid balance.

Use clear and simple language when communicating. Be patient, provide visual aids if necessary, and encourage non-verbal communication. Offer support and education to family members and caregivers. Providing respite care options can help prevent caregiver burnout. Discuss and document advance care directives with patients and their families, ensuring their wishes are known and respected. Incorporate regular, appropriate exercise into the routine to promote physical and mental well-being. Consult with healthcare professionals to determine suitable activities. Address any pain or discomfort promptly, as it can exacerbate behavioral symptoms in dementia patients. Regularly reassess the patient's condition and adjust the care plan as needed to accommodate changes in cognition, physical health, or emotional well-being. It's important to note that these recommendations should be tailored to each individual's unique needs and regularly reassessed as the patient's condition evolves. Consultation with healthcare professionals experienced in dementia care is crucial for effective management.

## Conclusions

In summary, our investigation marks the first comprehensive exploration of the disease burden among a substantial cohort of dementia patients in the Al-Baha Province of Saudi Arabia. This study offers valuable insights into the overall health status of these patients, serving as a significant reference for future comparative research. The findings hold the potential to raise awareness among clinicians and policymakers regarding the frequent occurrence of comorbidities in Parkinson's disease, Alzheimer's disease, or vascular dementia patients. This awareness could lead to enhancements in medical care and the quality of life for these individuals. Furthermore, recent suggestions linking systemic disorders with Amyloid beta metabolism in the brain and AD pathogenesis are noteworthy. Given the substantial burden of comorbidities in PD, AD, and VaD, addressing dysfunction in peripheral tissues and organs becomes crucial for effectively treating these types of dementia in the future.
